# Utilizing metagenomic next-generation sequencing and phylogenetic analysis to identify a rare pediatric case of *Naegleria fowleri* infection presenting with fulminant myocarditis

**DOI:** 10.3389/fmicb.2024.1463822

**Published:** 2024-11-13

**Authors:** Liangkang Lin, Lili Luo, Mei Wu, Jun Chen, Yi Liao, Haiyang Zhang

**Affiliations:** ^1^Department of Pediatrics, West China Second University Hospital, Sichuan University, Chengdu, China; ^2^Department of Pediatrics, The Eighth Affiliated Hospital, Sun Yat-sen University, Shenzhen, China; ^3^Key Laboratory of Birth Defects and Related Diseases of Women and Children, Ministry of Education, Chengdu, China; ^4^Department of Radiology, West China Second University Hospital, Sichuan University, Chengdu, China

**Keywords:** fulminant myocarditis, primary amoebic meningoencephalitis, metagenomic next-generation sequencing, *Naegleria fowleri*, phylogenetic analysis

## Abstract

**Background:**

*Naegleria fowleri* (*N. fowleri*), a rare and typically lethal amoeba, most commonly causes primary amoebic meningoencephalitis (PAM). This case report describes an exceptionally rare presentation of fulminant myocarditis as the primary manifestation in a 6-year-old child, diverging from the typical neurological pathogenesis associated with *N. fowleri* infection. Beyond neurological afflictions, the child developed arrhythmias and cardiac failure, necessitating treatment with extracorporeal membrane oxygenation (ECMO).

**Methods:**

Diagnosis was confirmed via metagenomic next-generation sequencing (mNGS) of both blood and cerebrospinal fluid (CSF). This analysis not only substantiated the infection but also revealed a potential new genotype of *N. fowleri*, designated k39_3, suggesting broader genetic diversity than previously recognized.

**Results:**

Immediate treatment with Amphotericin B (Am B) and rifampin was initiated upon diagnosis. Despite aggressive management and supportive care, the patient failed to maintain hemodynamic stability, continued to show a decrease in cardiac output, and exhibited relentless progression of central nervous system failure, culminating in death within 72 h.

**Conclusion:**

Our report documents a rare pediatric case of *N. fowleri* infection presenting with fulminant myocarditis, revealing an unexpected clinical manifestation and broadening the known spectrum of its effects. This emphasizes the need for enhanced surveillance and targeted research to understand the pathogenic mechanisms and improve treatment strategies.

## Background

*Naegleria fowleri* (*N. fowleri*) is a unicellular amoeba primarily found in warm freshwater habitats worldwide, including lakes, rivers, and hot springs (Maciver et al., 2020)^.^ Infection cases are exceedingly rare but associated with high fatality rates due to primary amoebic meningoencephalitis (PAM). *N. fowleri* is believed to invade the brain through olfactory neuroepithelium, leading to significant tissue destruction and inflammation. Despite ongoing research, specific pathogenic mechanisms driving PAM are not fully elucidated. It has been suggested that the pathogenesis of PAM involves the release of multiple enzymes and toxins by *N. fowleri* that directly disrupt neural tissue and induce an excessive host immune response, exacerbating tissue damage (Güémez and García, 2021).

In contrast to typical presentations of rapidly progressive PAM, this case report details a rare instance of *N. fowleri* infection manifesting initially as fulminant myocarditis. Confirmation of *N. fowleri* was achieved through metagenomic next-generation sequencing (mNGS) of both peripheral blood and cerebrospinal fluid (CSF). The complexity and severity of this case highlight the critical need for clinicians to remain alert to the possibility of rare pathogens like *N. fowleri* in instances of unexplained myocarditis, thereby facilitating timely and effective intervention.

## Case presentation

A six-year-old female child who was admitted to the pediatric intensive care unit (PICU) at West China Second University Hospital in April 2024 with symptoms of fever, headache, vomiting, and lethargy. Forty-eight hour prior to admission, she was diagnosed with acute upper respiratory tract infection and acute gastritis at a local hospital, where she was treated with oral cefaclor. Three hour prior to admission, she experienced a seizure lasting 5 min followed by persistent coma. The child had no history of recurrent respiratory infections, congenital heart disease, or any significant infectious diseases. She had swum in an indoor heated public pool 7 days before symptom onset.

**Figure 1:**
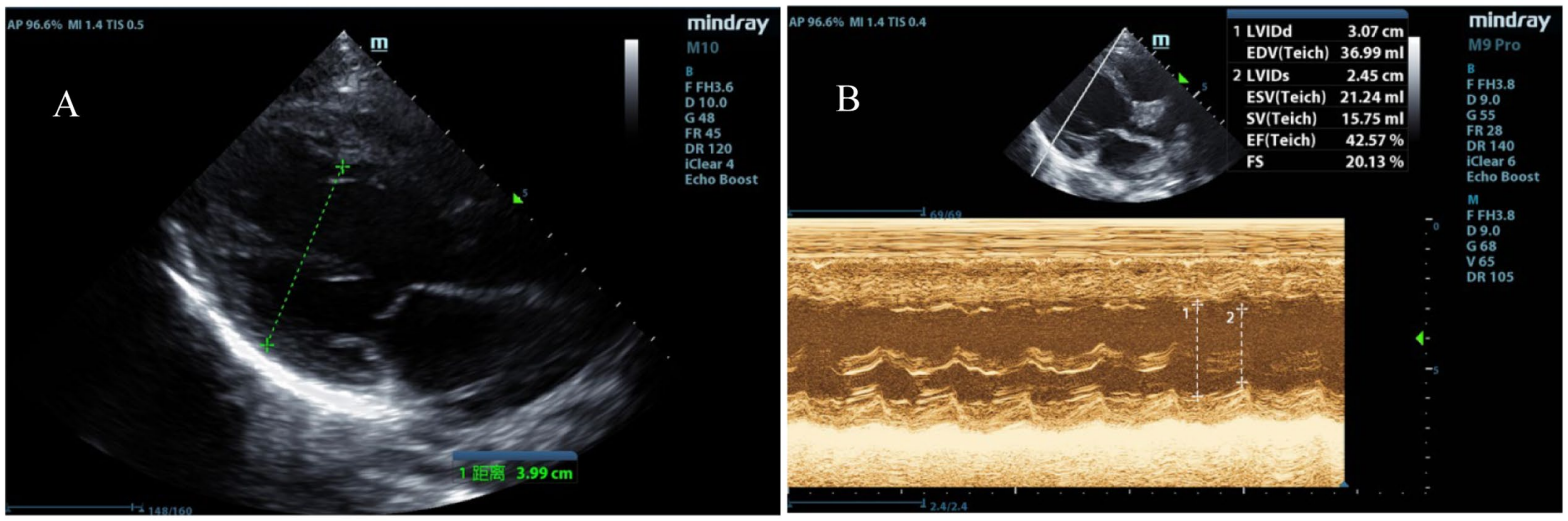
Echocardiographic indicators of left heart failure. (A) Enlargement of the left ventricular chamber. (B) Reduced left ventricular systolic function with an EF of 42% and FS of 20%.

The physical examination revealed a temperature of 39.2°C, a heart rate of 184 beats per minute, a respiratory rate of 39 breaths per minute, a blood pressure of 112/74 mmHg, and an oxygen saturation of 98% on 5 L/min of oxygen via face mask. The Glasgow coma scale (GCS) score was 8, indicating a severe impairment of consciousness (with a GCS score ≤ 10). The pupils were equal, round, 5 mm in diameter, and showed a sluggish reaction to light. She exhibited irregular breathing, a weak and rapid pulse, and diminished heart sounds. Neck stiffness and bilateral Babinski signs were positive; pathologic reflexes were absent. Laboratory findings included arterial blood gas analysis showing pH 7.527 (normal range: 7.35–7.45), pO_2_ 110.6 mmHg (normal range: 83–108 mmHg), pCO_2_ 19.9 mmHg (normal range: 35–45 mmHg), BE −4.0 mmol/L (normal range: −3.0-3.0 mmol/L), HCO^3−^ 16.5 mmol/L (normal range: 21.8–26.9 mmol/L), and lactate 1.75 mmol/L (normal range: 0.5–1.6 mmol/L). Procalcitonin was 3.68 ng/mL (normal range: <0.1 ng/mL). Complete blood count revealed white blood cells (WBC) of 13.0 × 10^9^/L (normal range: 4.0–11.0 × 10^9^/L), neutrophils 80.8% (normal range: 40–75%), platelets 434 × 10^9^/L (normal range: 150–400 × 10^9^/L), and hemoglobin 127 g/L (normal range: 120–160 g/L). C-reactive protein was 28.2 mg/L (normal range: <10 mg/L). Cardiac troponin I was 15.239 μg/L (normal range: 0.1–0.2 μg/L). Electrocardiogram showed sinus tachycardia with ST-segment alterations (elevation >0.1 mV in leads I and aVL, and depression between 0.05 and 0.2 mV in leads II, III, and aVF). Echocardiographic findings revealed left ventricular dilation and impaired systolic function, evidenced by an ejection fraction (EF) of 42% and fractional shortening (FS) of 20% (Figures 1A,B). Initial diagnoses included fulminant myocarditis, sepsis, heart failure, and cardiocerebral syndrome, based on clinical presentation, laboratory findings, and imaging results.

Upon admission, immediate intubation and invasive mechanical ventilation were initiated. Hypothermia therapy for the brain, empirical broad-spectrum antibiotic treatment with meropenem and vancomycin, antiviral therapy with acyclovir, inotropic support with epinephrine and dobutamine, intracranial pressure management with mannitol, immunoglobulin infusion, and cardioprotection were administered. Peripheral blood was sent for mNGS. Five hours post-admission, the patient’s blood pressure dropped to 75/48 mmHg. Reevaluation of arterial blood gases indicated severe metabolic acidosis and elevated lactate levels reaching 12 mmol/L. Despite increased epinephrine doses, blood pressure remained unstable. Given the persistent hemodynamic failure despite high doses of vasoactive drugs, bedside veno-arterial extracorporeal membrane oxygenation (ECMO) was initiated right internal jugular vein incision and artery incision, with initial settings of a centrifugal pump speed of flow rate of 1.5 L/min, FiO2 100%, and sweep gas flow of 2 L/min. Eighteen hours into admission, severe metabolic acidosis was confirmed. Sodium levels were critically high at 170 mmol/L, with potassium at 2.2 mmol/L, calcium at 1.5 mmol/L, and chloride over 140 mmol/L. Due to the severe internal environment disorder, continuous renal replacement therapy (CRRT) was integrated into the ECMO circuit in the continuous vein–vein hemodialysis (CVVHD).

**Figure 2:**
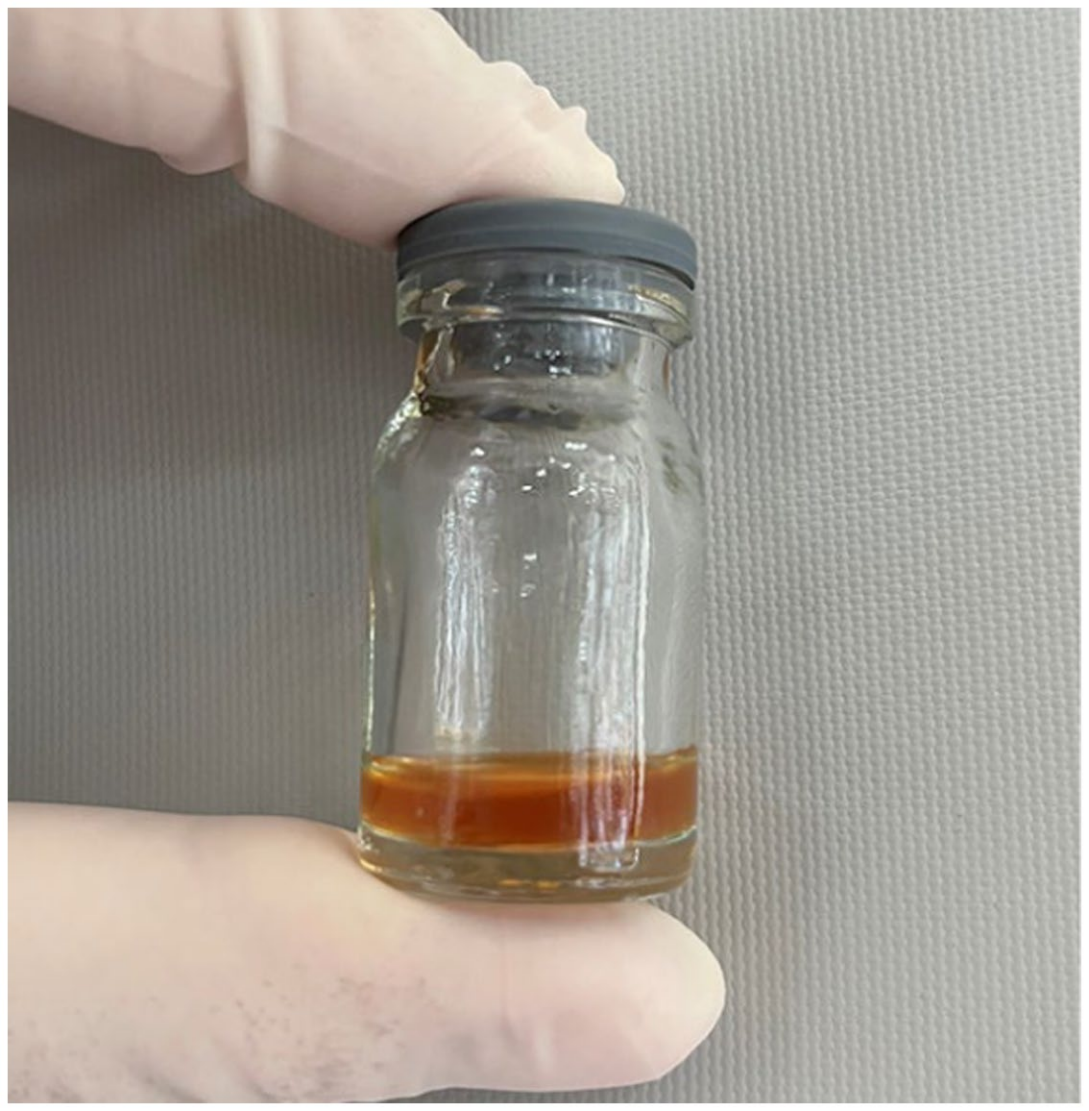
Lumbar puncture revealed viscous purulent CSF with a pressure of about 80 mmH_2_O.

**Figure 3:**
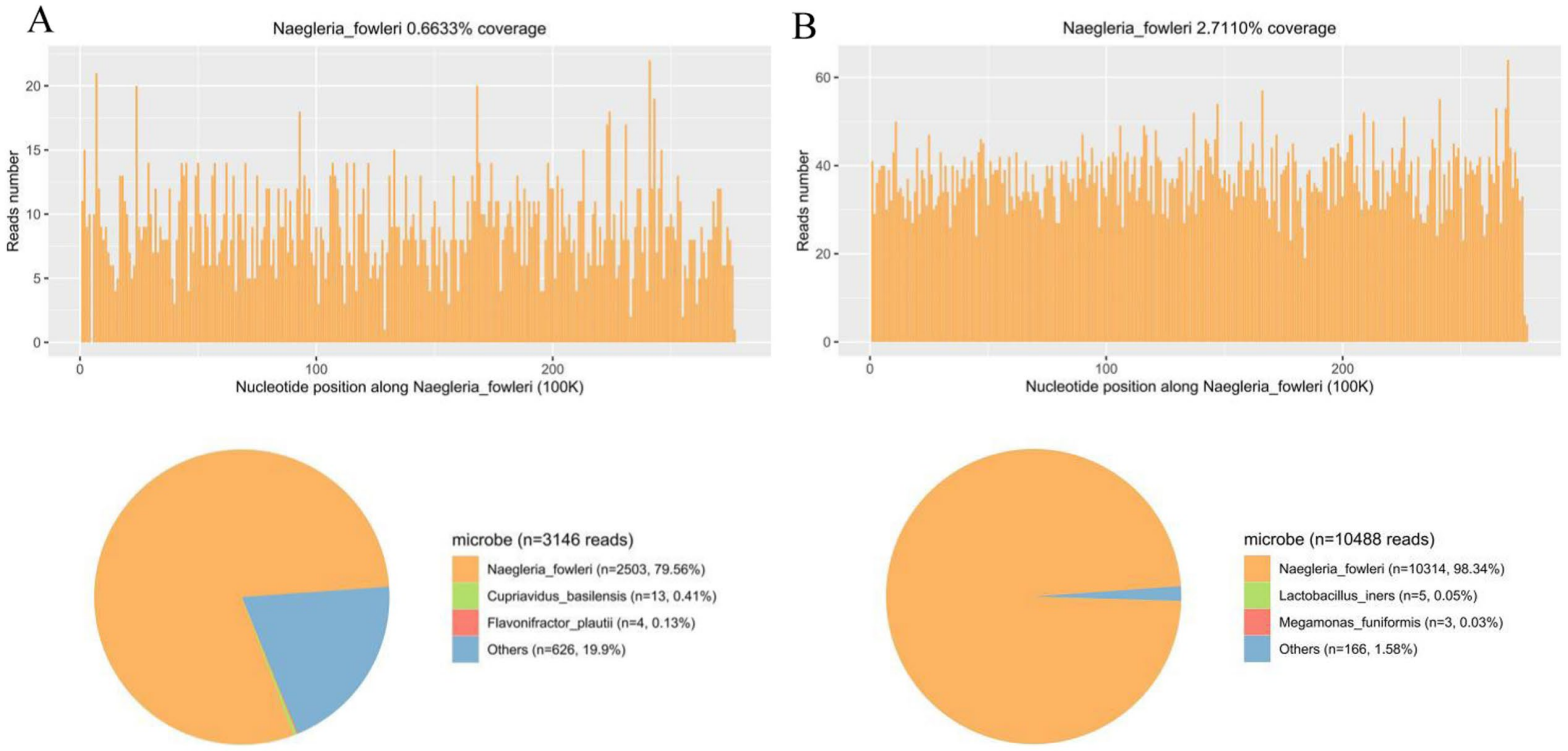
mNGS analysis of peripheral blood and CSF samples for *N. fowleri* Detection. (A) The coverage and abundance of *N. fowleri* detected by mNGS using blood sample. The others section of the pie chart displayed 626 reads, constituting 19.9% of the total, which included *Nocardia, Cupriavidus*, and *Akkermansia*, identified as clinically insignificant background flora. (B) The coverage and abundance of *N. fowleri* detected by mNGS using CSF sample. The others section of the pie chart displayed 166 sequences, constituting 1.58% of the total, which included *Anaerococcus*, *Atopobium*, and *Actinomyces*, identified as clinically insignificant background flora.

**Figure 4:**
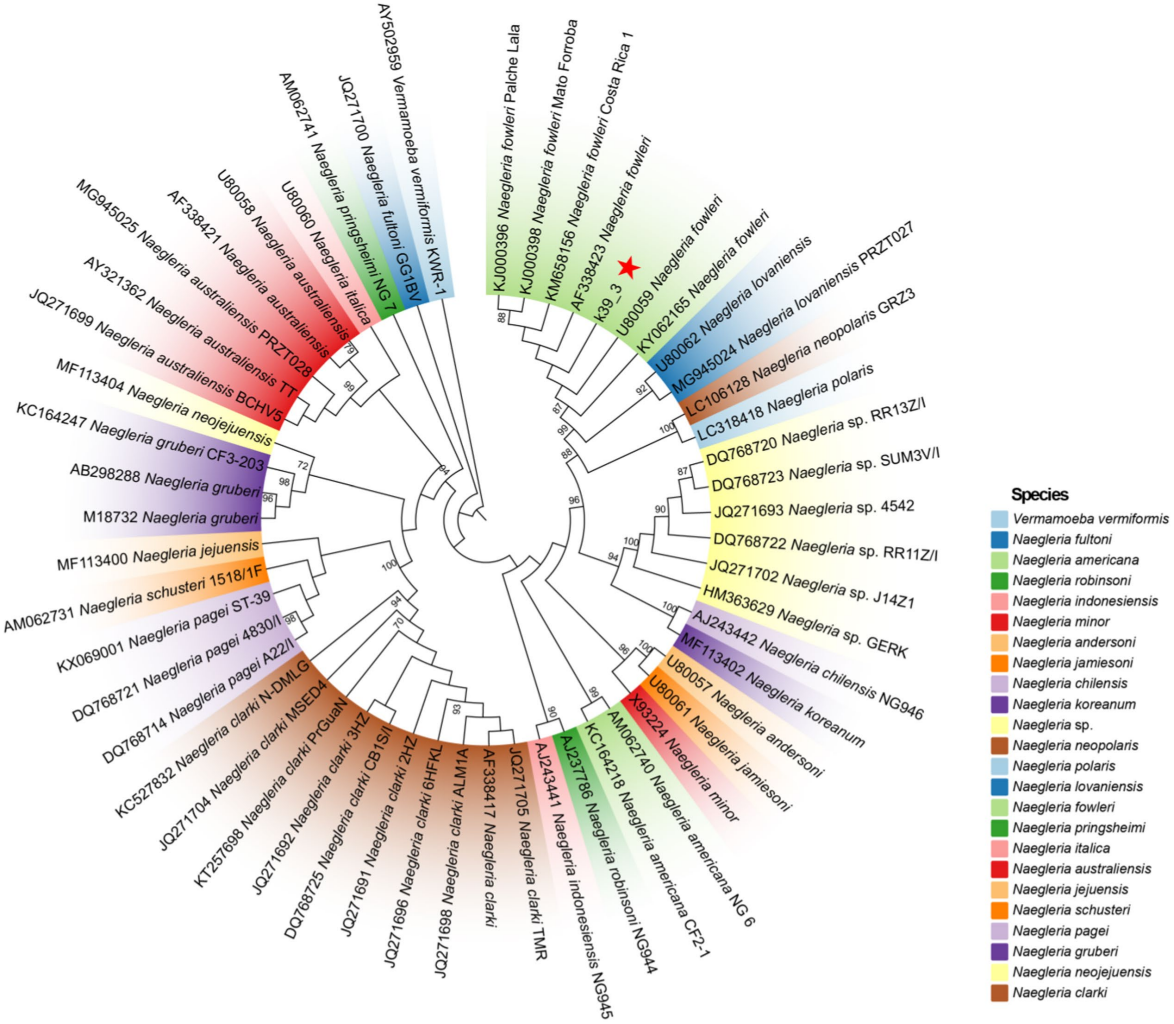
The ML phylogenetic tree demonstrates that contig k39_3 is classified within the *N. fowleri*. A total of 23 species and 53 distinct genotypes were included in the *Naegleria* spp. analysis. The ML phylogenetic tree reveals significant branch support, with bootstrap confidence values exceeding 90%, indicating a high level of reliability in the inferred relationships among the taxa.

**Figure 5:**
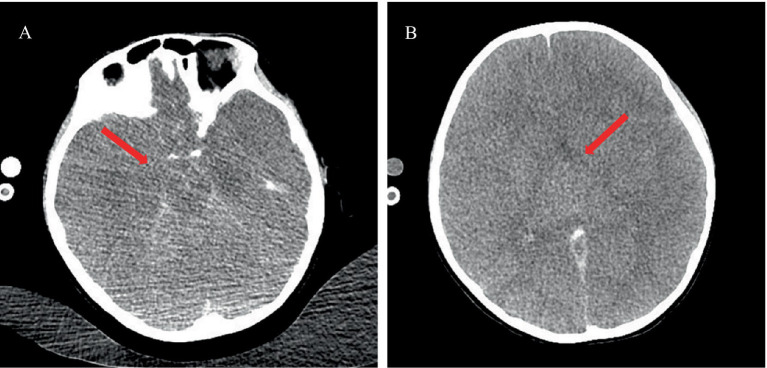
Cranial CT imaging showing herniation. (A) Cranial CT at the level of the cisterns indicating closure of the cisterns (red arrow). (B) Cranial CT at the level of the basal ganglia demonstrating narrowed lateral ventricles, suggestive of herniation (red arrow).

**Figure 6:**
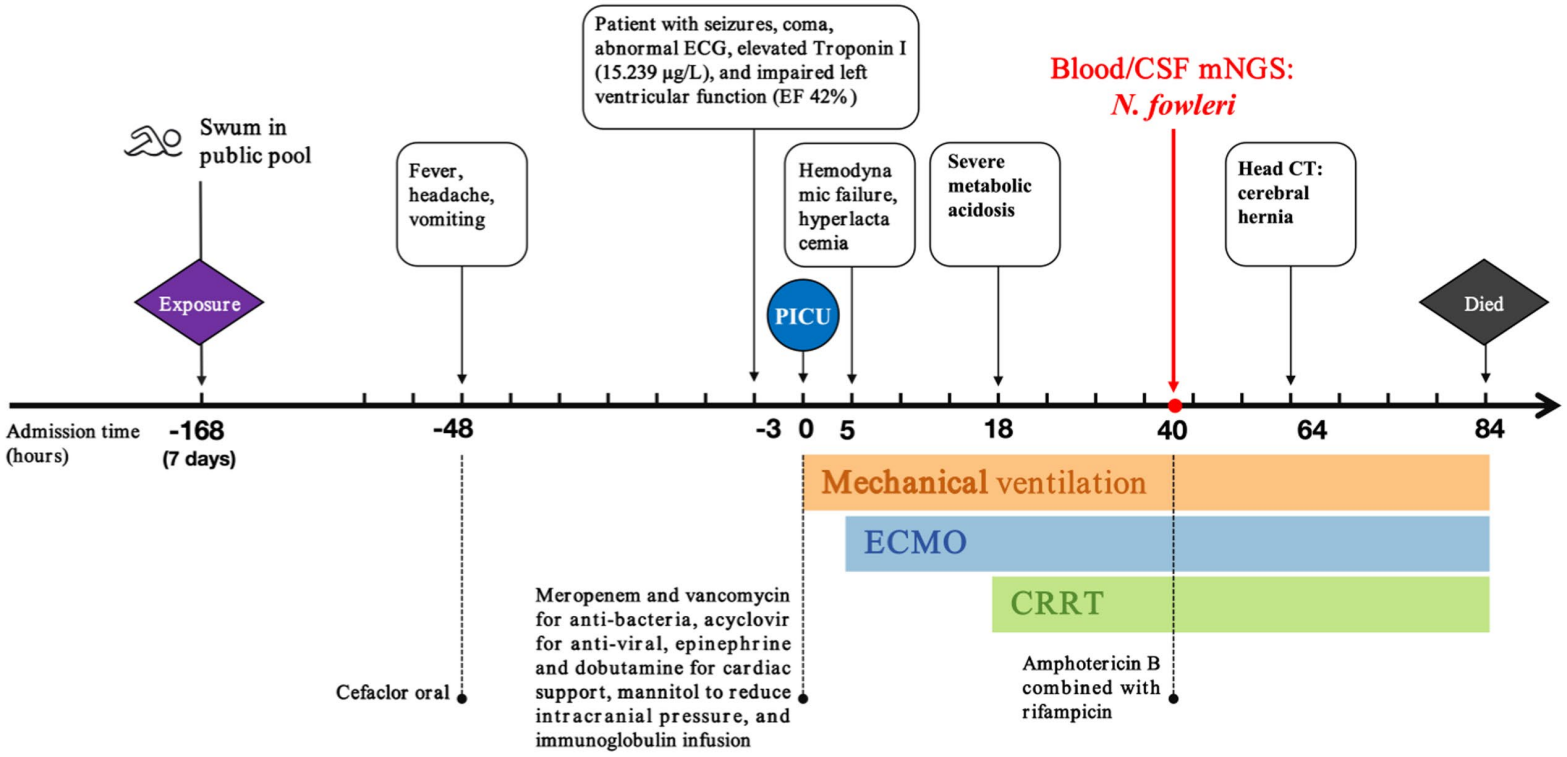
The timeline of disease progression and treatment.

Lumbar puncture revealed viscous purulent CSF with a pressure of about 80 mmH_2_O (Figure 2). Upon cytological examination of the CSF, a marked infiltration of white blood cells was observed. CSF analysis showed red blood cells (RBC) 3,200 × 10^6^/L (normal reference value: 0), WBC 960 × 10^6^/L (normal range: 0–15 × 10^6^/L), protein concentration approximately 10,000 mg/L (normal range: 80–430 mg/L), glucose 4.76 mmol/L (normal range: 2.8–4.5 mmol/L), chloride 123.2 mmol/L (normal range: 120–130 mmol/L), lactate dehydrogenase (LDH) 2,945 U/L (normal range: 5–35 U/L), and lactate 7.8 mmol/L (normal range: 1.0–2.8 mmol/L). At 40 h post-admission, blood mNGS identified *N. fowleri* with high confidence and specificity (sequence 2,503 reads, relative abundance 79.56%, Figure 3A). CSF mNGS also indicated *N. fowleri* (sequence 10,314 reads, relative abundance 98.34%, Figure 3B). Phylogenetic analysis suggested a potentially novel *Naegleria* species k39_3 (Figure 4). These findings led to the diagnosis of PAM, prompting the addition of Amphotericin B (Am B) and rifampicin to the treatment regimen. Furthermore, culture and PCR analyses of blood and CSF yielded negative results for other pathogens despite clinical evidence of infection. Insufficient sample volume precluded successful CSF smear testing. Sixty-four hours after admission, subsequent imaging and multimodal central nervous system monitoring revealed severe brain dysfunction. Head CT findings indicated effacement of the sulci and narrowing of the lateral ventricles, suggestive of cerebral herniation (Figures 5A,B). Diffuse slow waves on an 8-h EEG, significantly reduced cerebral blood flow on transcranial Doppler, and regional oxygen saturation as low as 10% on near-infrared spectroscopy (NIRS). Neurosurgical consultation concluded that decompressive craniectomy offered minimal benefit for this patient due to the severe concurrent cardiac and cerebral failure, indicating a poor prognosis even with potential surgical relief of intracranial pressure. Despite continued critical care, the patient’s cardiac function failed to recover, and the comatose state was irreversible. Eighty-four hours after admission, the family opted to withdraw care, and the child subsequently passed away. The timeline of treatments is displayed in Figure 6.

## Methods

The mNGS test was performed first. Samples included 2 mL each of peripheral blood and CSF, preserved in Cell-Free DNA BCT STRECK tubes and maintained at 6–35°C for analysis. DNA was isolated from 200 μL of plasma using the QIAGEN QIAamp DNA Micro Kit, with quality assessed via Qubit fluorometry and agarose gel electrophoresis. Libraries were prepared by end-repairing DNA, utilizing the QIAseq™ Ultralow Input Library Kit, and evaluated similarly. Sequencing was conducted on an Illumina NextSeq 550 (Illumina, San Diego, USA), using a 75-bp single-end shotgun metagenomic approach. After sequencing, data underwent quality filtering to remove adapters, low-quality and low-complexity sequences, and reads aligned to the human genome using SNAP software. Remaining reads were aligned to a microbial database from NCBI containing over 20,000 microorganism genomes. This comprehensive genomic analysis set the stage for the subsequent phylogenetic exploration of the unique *Naegleria* sequences.

Following the identification of *N. fowleri* in the CSF of this patient via mNGS, we proceeded to construct a phylogenetic tree to further analyze the strain. Quality control of sequencing reads from both blood and CSF samples was performed using Fastq, with subsequent alignment against the human reference genome (hg38) utilizing Bowtie2. Non-human reads were mapped to the *N. fowleri* reference genome (GCA_014843625.1) employing the Burrows-Wheeler Aligner (BWA). Mapped reads were then assembled into contigs (k39_1, k39_3, k39_4) via Megahit. Of these, contig k39_3, which was identified to align with the 18S rRNA gene of *N. fowleri* according to NCBI BLAST, was selected for detailed phylogenetic analysis. A phylogenetic tree was constructed using the Maximum Likelihood (ML) estimation method in IQ-TREE. This analysis employed the Generalized Time Reversible model with nucleotide frequency, invariable sites, and rate variation across sites (GTR + F + I + R2), selected based on the Akaike Information Criterion (AIC). A comprehensive set of 10,000 Ultrafast bootstrap replicates was executed to validate the classification of the k39_3 strain within the *Naegleria* spp. lineage. For visualization, the tree was depicted in Interactive Tree Of Life (iTOL).

## Discussion

*Naegleria fowleri* is one of the few free-living amoebae (FLA) capable of causing fatal infections in humans, with an incubation period typically ranging from 5 to 7 days (Güémez and García, 2021). Healthy children and young adults are particularly susceptible to PAM following exposure to water contaminated with *N. fowleri*, leading to an extremely high mortality rate (Król-Turmińska and Olender, 2017). The actual incidence of PAM may be underestimated due to frequent misdiagnosis or non-reporting, a situation exacerbated by a general lack of awareness. Often, routine clinical evaluations fail to detect its hallmark symptoms, and CSF analyses mimic those of bacterial meningitis—showing elevated leukocyte counts, increased protein levels, and altered glucose concentrations. Although early neuroimaging might appear normal, the disease’s progression can reveal distinct brain damage or hemorrhage (Shaukat et al., 2024). Most diagnoses are made post-mortem due to rapid disease advancement, non-specific symptoms, and limited diagnostic tools. Autopsies typically show softening and swelling of cerebral tissues, focal demyelination, and inflammation in both the olfactory bulbs and, in cases like this involving myocarditis, cardiac tissues. Enhanced imaging techniques and cardiac biomarkers should be considered in acute settings when *N. fowleri* exposure is suspected. The meninges (arachnoid and piamater) are congested and diffusely infiltrated (Zhang and Cheng, 2021). The mortality rate for PAM is up to 98%, with fewer than ten known survivors to date (Haston and Cope, 2023). This high mortality rate is attributed to delayed diagnosis, the absence of safe and quick effective treatments, and challenges in drug delivery to the brain.

Upon inhalation of *N. fowleri*-contaminated water, the parasite adheres to and breaches the nasal mucosa, subsequently ascending via the olfactory nerves through the cribriform plate to reach the olfactory bulbs in the brain. It then progresses, causing cerebral proliferation, edema, and herniation, with majority fatal results. The amoeba’s pathogenesis involves tissue phagocytosis and cytolytic molecule secretion, damaging host cells (Sohn et al., 2019). Adhesion of *N. fowleri* to host cells activates signal pathways, upregulating proteins and proteases essential for central nervous system (CNS) invasion and proliferation (Jamerson et al., 2017; Jamerson et al., 2012). This interaction induces the release of lytic enzymes such as neuraminidase and phospholipases, causing neural damage, apoptosis, and severe immune responses that exacerbate CNS injury (Lê et al., 2022). Recent *in vitro* studies using murine BV-2 microglial cells demonstrated that *N. fowleri* excretory-secretory proteins (NfESPs) trigger specific inflammatory responses in these cells. Upon exposure to NfESPs, BV-2 cells become activated, leading to the enhanced secretion of chemotactic and pro-inflammatory cytokines, including IL-1*α* and TNF-α. This activation contributes to the overall inflammation observed in the central nervous system during *N. fowleri* infection. The pathogenic effects of NfESPs involve both direct cytolytic damage to host neuronal cells and the induction of inflammatory immune responses (Lee et al., 2017).

Huang et al. recently described a case of suspected PAM caused by *N. fowleri*, which was complicated by myocarditis (Huang et al., 2024). Their patient presented with myocarditis symptoms alongside typical PAM features, including persistent tachycardia, right bundle branch block, and echocardiographic evidence of heart failure, ultimately leading to death from cardiac arrest. In contrast, our case includes a more comprehensive account of therapeutic interventions and clinical management, aspects that were notably absent in Huang et al.’s report. The overlapping myocarditis symptoms in both cases underscore the necessity of recognizing cardiac involvement in *N. fowleri* infections. In our case, due to the unavailability of an autopsy on the patient, direct evidence of *N. fowleri*, in the heart is not ascertainable. From the progression of the case, we speculate that *Naegleria* spp. could potentially induce myocarditis through alternative mechanisms, as indicated in existing literature. Martinez AJ’s review has delineated the pathophysiological pathways by which FLA infections may precipitate arrhythmia and myocardial necrosis (Martinez and Visvesvara, 1991). Building upon these insights, we investigated diverse potential etiologies and pathogenic mechanisms contributing to myocarditis in our case. These include pre-existing cardiac conditions, concurrent non-amebic infections, and electrolyte imbalances, among others. Particularly, the absence of known cardiac conditions and the acute presentation suggest that the myocarditis may primarily be due to direct or toxin-mediated invasion by *N. fowleri*, exacerbated by subsequent metabolic derangements such as acidosis and electrolyte imbalances. Metabolic acidosis, particularly anionic (lactic) and hyperchloremic forms, significantly impacts cardiac function by reducing cellular pH, which in turn impairs myocardial contractility and electrical stability, contributing to arrhythmogenesis. Acidosis triggers excessive catecholamine release, causing tachycardia due to increased hydrogen concentration and, in some cases, ventricular arrhythmias; severe pH reductions inhibit catecholamine production, leading to bradycardia, reduced myocardial contractility, and hypotension (Stewart et al., 1965; Mitchell et al., 1972). Studies indicate that extracellular acidosis alters potassium channel mechanisms in coronary artery smooth muscle cells, reducing membrane excitability, thereby changing impulse propagation and causing ventricular arrhythmias in the setting of intact myocardium (Nagai et al., 2010). Additionally, hypokalemia-related myocardial fiber necrosis is associated with fiber rupture and necrosis with predominantly lymphocytic and occasional fibroblast infiltration (Leoni and Rello, 2017). Our patient exhibited severe metabolic acidosis and electrolyte disturbances, for which we promptly utilized CRRT to correct these issues. In cases of ischemia, myocardial changes such as cloudy swelling, loss of striations, and interstitial edema can appear as early as 30 min after permanent coronary artery occlusion in dogs. These early changes are reversible within 60 min if blood flow is restored. However, prolonged ischemia leads to myocardial necrosis characterized by infiltration of polymorphonuclear leukocytes and macrophages after 12 h (Shnitka and Nachlas, 1963). Thus, hypoxia likely played a role in our patient’s myocardial pathology. However, detailed reports of such processes in cases of *N. fowleri* infection are lacking. Focal myocardial lesions may be caused by nonspecific stress. Current research shows that high doses of catecholamines can impact the heart, suggesting that brain infection, fever, and hypoxia may release significant amounts of sympathetic amines, in turn exacerbating hypoxia-induced cardiac damage, ultimately leading to fulminant myocarditis. Toll-like receptors (TLRs), involved in responses to various parasites and expressed in epithelial, endothelial, and other cardiovascular cells, provide short-term cardiovascular protection when activated transiently but can induce chronic inflammation when overactivated (Vallejo, 2011; de Kleijn and Pasterkamp, 2003). Upon exposure to inflammatory stimuli, myocardial cells secrete both pro-inflammatory and anti-inflammatory cytokines, which initiate and modulate the inflammatory response. Additionally, they release chemokines that recruit and activate appropriate inflammatory cells (Boyd et al., 2006). It should be noted, however, that the heart is not a typical site of *N. fowleri* infection, and TLRs research is limited to animal models of *Acanthamoeba* infection (Kot et al., 2020).

For diagnosing *N. fowleri* infections, CSF smear and brain tissue biopsy remain the primary methods (Berger, 2022). To establish a culture of *N. fowleri*, CSF samples should be collected using aseptic techniques and stored at room temperature (15–25°C). These conditions facilitate the survival of the thermophilic amoebae, allowing for the direct observation of live amoebae under microscopy and growth in appropriate culture media. However, due to the rapid progression of the disease, children often die before a definitive diagnosis is made. Historically, many cases of PAM were confirmed post-mortem (Kofman and Guarner, 2022). Unfortunately, the patient ceased treatment and rapidly succumbed. CSF culture yielded negative results, and due to the family’s refusal of autopsy, direct evidence of *N. fowleri* in brain tissue could not be established. Nevertheless, advancements in molecular diagnostic techniques have greatly enhanced efficiency. PCR and isothermal DNA amplification have been developed for the specific identification of *N. fowleri* in clinical and environmental samples, improving diagnostic speed and accuracy (Xue et al., 2018; Mahittikorn et al., 2015). Additionally, cutting-edge mNGS is thriving. This technology provides annotated genomic sequences that offer crucial insights into the pathogenicity and virulence of the organisms. Wang Q previously reported the first case of PAM in mainland China (Wang et al., 2018). Similar to our case, this case identified a high number of *N. fowleri* sequences with high confidence in CSF through NGS, despite negative culture results, confirming PAM. This highlights NGS as a rapid and accurate diagnostic tool for *N. fowleri*, unaffected by limitations of conventional techniques like culture, PCR, and smears. Additionally, our investigation revealed confident *N. fowleri* sequences in peripheral blood via NGS, suggesting potential extracerebral dissemination or systemic removal of pathogen excreted virulance factors or biomarkers of PAM disease. This finding extends the understanding of *N. fowleri* infection dynamics beyond CSF involvement, further supporting the diagnostic evidence in our patient. NGS of environmental and clinical strains could help develop more specific genotyping tools for tracing the sources of infection in clinical cases of *N. fowleri* and conducting molecular epidemiological studies. Moreover, constructing phylogenetic trees based on mNGS genomic data can facilitate a deeper understanding of these rare pathogens. Phylogenetic analysis in this case reveals that the k39_3 genotype clusters closely with previously reported genotypes of *N. fowleri*. This suggests that k39_3 may represent an unrecognized genetic variant, characterized by distinct genetic markers while still classified within the *N. fowleri* species. *N. fowleri* typically causes PAM, but the association with myocarditis in this case could be linked to specific genetic factors inherent to the k39_3 genotype. This genotype could potentially possess unique virulence factors or gene expressions that may facilitate crossing the blood–brain barrier and triggering severe inflammatory responses in the cardiac tissue. Alternatively, the novel genotype might influence host immune responses differently, potentially exacerbating cardiac damage. A deeper understanding of these mechanisms will require extensive genomic analysis and functional studies to identify and characterize specific gene products of k39_3 that contribute to its pathogenicity.

Due to the rarity and high mortality rate of *N. fowleri* infections, treatment poses significant challenges. Current knowledge about potential therapies is derived from case reports and various *in vitro* and *in vivo* studies. Am B, the most commonly used antifungal in the treatment of PAM, disrupts the cell membrane of amoebae, leading to apoptosis-like programmed cell death (PCD) (Cárdenas-Zúñiga et al., 2017). However, the effectiveness of Am B is limited when administered intravenously due to its poor penetration of the blood–brain barrier and associated systemic toxicity. Alternate administration routes, such as intraventricular delivery via an Ommaya reservoir or intrathecal administration through lumbar or ventricular drains, have been explored to enhance drug delivery to the central nervous system (McLaughlin and O'Gorman, 2019; Saleem et al., 2009). Although rifampin can achieve therapeutic concentrations in the central nervous system as monitored via CSF levels, studies by Ondarza et al. indicate a lack of evidence supporting the efficacy of standard doses in treating PAM (Sullins and Abdel-Rahman, 2013; Nau et al., 1992; Thong et al., 1977; Ondarza et al., 2006). There are currently no *in vitro* studies or clinical guidelines recommending specific dosages or courses of treatment for Am B and rifampin. Miltefosine (MLT) is an alkylphosphocholine compound employed in the treatment of breast cancer and leishmaniasis. Notably, it has been reported to successfully treat a 12-year-old girl with PAM, achieving full neurological recovery, thereby highlighting its potential as a novel therapeutic for PAM (Heggie and Küpper, 2017). Controlled *in vitro* studies have shown that azole derivatives possess pronounced anti*-N. fowleri* activity, often surpassing that of MLT. Specifically, itraconazole and posaconazole have shown exceptional potency with minimal effective concentrations (EC50) ≤ 0.01 μM, proving more effective than Am B (Debnath et al., 2017). The Centers for Disease Control and Prevention (CDC) recommends a regimen that includes AmB, rifampin, azithromycin, MLT, and either fluconazole or ketoconazole (Cope and Ali, 2016). Research by Colon et al. found that posaconazole inhibits the growth of *N. fowleri* within the first 12 h of exposure and enhances therapeutic outcomes when combined with azithromycin (Colon et al., 2019). Moreover, the sterol pathway can be blocked by isavuconazole, ebastine, and tamoxifen, which inhibit CYP51, sterol C24-methyltransferase (SMT), and sterol 87-isomerase (ERG2), respectively (Zhou et al., 2018). A combination of these drugs showed synergistic effects *in vitro* against *N. fowleri*, suppressing its growth by 95% within 48 h post-treatment (Zhou et al., 2018). Thus, inhibiting two enzymes involved in the sterol pathway may be more efficacious than targeting a single enzyme. High-throughput phenotypic screening of the Calibr ReFRAME library by Rice et al., which includes over 12,000 compounds, identified 19 compounds capable of inhibiting *N. fowleri in vitro* within 24 h (Rice et al., 2020). Given the known mechanisms of many repurposed drugs, these compounds are promising for validating new targets through structure-based drug design.

Ongoing research aims to identify safer and more effective treatments for *N. fowleri* infections. The poor permeability of the blood–brain barrier has recently brought nanoparticle technology to the forefront of pharmaceutical interest due to its potential to enhance drug efficacy and reduce dosage requirements. Nanoparticle drug delivery systems, by increasing bioavailability, reducing cytotoxicity, and achieving site-specific targeting, offer promising avenues for enhancing the delivery of therapeutic agents directly to the central nervous system in *N. fowleri* infections. Studies have shown that conjugating drugs with nanoparticles, particularly silver nanoparticles combined with anti-amoebic medications, enhances the stability and bioavailability of these drugs (Rajendran et al., 2017; Rajendran et al., 2020). It is anticipated that further advancements in drug therapies and delivery systems will be utilized in the future.

We report the second documented instance of myocarditis linked to *Naegleria* spp., following a previous report by Huang et al. (2024). This case involves a child with myocarditis linked to a novel *N. fowleri* genotype, identified as k39_3 through phylogenetic analysis. Limitations arise from the single-case basis of this study and the inability to perform whole-genome sequencing (WGS) due to limited samples, restricting precise evolutionary classification and broader representation. Moreover, while the association between myocarditis and the detected *N. fowleri* is suggestive, the lack of direct evidence of *N. fowleri* in myocardial tissues raises important concerns regarding the speculation of extracerebral dissemination. As we could not isolate clinical specimens, this hypothesis remains unsupported by direct evidence. It is plausible that the damage observed in extraneural organs may primarily result from a severe inflammatory response leading to a cytokine storm rather than direct parasitic invasion. Additionally, recent foundational studies are exploring the mechanisms of *N. fowleri* invasion beyond the central nervous system. For instance, Russell et al. identified small RNAs from *N. fowleri* in experimentally infected mice and subsequently in patient serum, revealing significant differences across various specimens (Russell et al., 2024). This line of research may pave the way for future identification and isolation of clinical samples, providing crucial insights into the pathogenesis of *N. fowleri* infections and their potential extracerebral effects. The FLA community is always trying to obtain newer or more recent clinical isolates to be able to characterize and perform drug sensitivity testing. Future studies should aim to clarify biochemical mechanisms and validate k39_3’s classification through phenotypic characterization and experimental models. Further research into *Naegleria*’s pathogenic pathways, particularly the k39_3 strain, could elucidate its potential link to increased myocarditis susceptibility and contribute to better prevention and treatment strategies for infections by waterborne pathogens.

## Conclusion

*Naegleria fowleri*, a rare but deadly free-living amoeba, remains enigmatic and under-researched, primarily due to the covert and rapid progression of its infections. Recent advancements in detection technologies have significantly expedited the identification of pathogens, including FLA. However, while these technological strides contribute to quicker diagnoses, the improvement in treatment outcomes for such infections remains variable and, unfortunately, relatively infrequent. Beyond the classic neurological manifestations, we report the notable case of fulminant myocarditis linked to *N. fowleri*, highlighting a previously unrecognized complication and expanding our understanding of its clinical spectrum. This underscores the potential expanding challenge of amoebic infections, ringing an alarm for heightened vigilance. Future research should concentrate on detailed molecular and cellular studies to elucidate the biological mechanisms through which *N. fowleri* contributes to myocarditis, with the aim of uncovering novel therapeutic targets and improving clinical management strategies.

## Data Availability

The datasets presented in this study can be found in online repositories. The names of the repository/repositories and accession number(s) can be found in the article/Supplementary material.
